# Posterior Reversible Encephalopathy Syndrome in a Patient With Macrophage Activation Syndrome and Herpes Simplex Virus 2

**DOI:** 10.7759/cureus.17273

**Published:** 2021-08-18

**Authors:** Joshua D Luster, Ricardo Galindo

**Affiliations:** 1 Medicine, Brooke Army Medical Center, San Antonio, USA

**Keywords:** posterior reversible encephalopathy syndrome (pres), hemophagocytic lymphohistiocytosis (hlh), herpes simplex virus 2, anakinra, macrophage activation syndrome

## Abstract

Posterior reversible encephalopathy syndrome (PRES) is a symptomatic and radiographic syndrome characterized by neurologic changes and concomitant neuroimaging findings typically involving posterior cerebral white matter edema. While there are many competing views on the pathophysiology of PRES, inflammatory conditions, particularly autoimmune diseases, tend to have some degree of correlation. Most cases where patients who have PRES and autoimmune diseases typically involve systemic lupus erythematosus. There is relatively little information in the literature on PRES occurring in adult patients with hemophagocytic lymphohistiocytosis (HLH)/macrophage activation syndrome or of concomitant herpes simplex virus 2 (HSV2) infection. Here, we present the case of a patient with PRES in the setting of adult-onset Still’s disease and HLH with concomitant HSV2 infection.

## Introduction

Posterior reversible encephalopathy syndrome (PRES) was described in the literature by Hichey and colleagues in 1996 with a series of 15 patients. They described 15 patients with headaches, seizures, visual disturbances, and other neurologic deficits and coined the term PRES. Two major theories have been proposed regarding the pathophysiology and development of PRES. The first involves hypertension and the breakdown of the autoregulation of the brain and vascular systems. This theory postulates that the blood pressure of patients fluctuates that overcomes autoregulation, which causes hyperperfusion, vascular leakage, and the development of vasogenic edema, leading to the neurologic symptoms seen in PRES. Posterior circulation is more often affected due to the relative decrease in sympathetic innervation which implies that posterior circulation has a relative decreased ability to autoregulate during blood pressure fluctuations. However, going against this theory, up to 30% of patients do not present with hypertension at the onset of neurologic symptoms and PRES has also been reported in anterior circulation [1]. The second theory proposes endothelial dysfunction as a secondary finding in association with other primary concerns. The proposed primary concerns include (pre)eclampsia, sepsis, immunomodulatory or cytotoxic agents, or autoimmune disorders. Due to the lack of evidence of hypertension at the onset of PRES, there has been a shift toward the second theory involving endothelial dysfunction.

While the theories causing the pathophysiology of PRES remain controversial, many factors correlate with the onset of PRES. Up to 45% of patients have an underlying autoimmune disease, most commonly systemic lupus erythematosus (SLE) [2]. It is believed that either autoimmune diseases or the treatment of autoimmune diseases, mostly associated with tacrolimus or cyclophosphamide, can be damaging to the endothelium, leading to dysfunction and subsequent vasogenic edema [2-5]. Other causes include renal failure which has been found in up to 55% of patients with PRES and is thought to lead to blood pressure fluctuations as well as endothelial damage and dysfunction [6]. Another cause is (pre)eclampsia as it can lead to both blood pressure fluctuations and endothelial dysfunction.

While there is no consensus on the definition or guidelines for the diagnosis of PRES, patients should present with neurologic symptoms, blood pressure fluctuations, vasogenic edema on imaging, and clinical suspicion for PRES. Some of the neurologic symptoms that can be useful for PRES include seizures, headaches, hallucinations, cortical blindness, paresthesias, or motor findings. Some studies have reported that seizures can be the primary neurologic sign and general tonic-clonic (GTC) seizures can occur in 60-75% of patients. Moreover, 10-15% of patients who present with GTC will likely develop status epilepticus. While PRES is often considered reversible and the prognosis is favorable with most patients making a full recovery in the first few weeks, not all patients fully recover and some have lifelong epilepsy, motor deficits, or other focal neurologic symptoms [1,6].

While PRES is typically thought of as being posteriorly dominant, there have been several studies demonstrating that this is not the case. There are three major subtypes of patterns seen on imaging to describe PRES. The most dominant is the parietal-occipital pattern that affects mainly the parietal and occipital lobes and is seen in 27.9% of patients with PRES [7]. The superior frontal sulcus pattern is seen in 27.2% of patients and primarily shows vasogenic edema around the superior frontal sulcus [7]. The last is the holohemispheric watershed pattern seen in 22.8% of patients with vasogenic edema across the frontal, parietal, and occipital lobes at the junctions of the anterior cerebral artery (ACA)/posterior cerebral artery (PCA) and middle cerebral artery (MCA) watershed areas [7]. Most patients present with a combination of these subtypes, with 98% showing occipital or parietal findings, 68% showing frontal findings, and 40% showing inferior temporal findings [7]. Magnetic resonance imaging (MRI) is superior compared to computed tomography (CT), with 45% of patients showing CT findings consistent with PRES, 33% showing nonspecific findings, and 22% showing normal imaging. While it has not been effectively studied, there have been suggestions that diffusion-weighted imaging (DWI) and apparent diffusion coefficient changes on MRI can be associated with poorer prognosis due to evidence of ischemia versus microhemorrhages.

The mainstay for the treatment of PRES is the reduction of blood pressure by 25% in the first four to six hours, along with preventing fluctuations of blood pressure. For patients who prevent with GTCs or other seizures, antiepileptic drugs (AEDs) are typically administered, although there is no consensus on the best or most effective AED for this patient population. Although lifelong AEDs for patients with GTCs or seizures caused by PRES are not required, some patients will have lifelong epilepsy and may require AEDs. The discontinuation of immunomodulators or immunosuppressives is controversial, but some studies have suggested that discontinuation may be detrimental. Studies have reported that the continuation of immunomodulators or immunosuppressives is beneficial to treat the underlying autoimmune disorder which is thought to be the driving factor in the development of PRES [3-5].

Hemophagocytic lymphohistiocytosis (HLH) is a rare and aggressive life-threatening overactivation of the cytotoxic T-cells and antigen-presenting cells [8]. Macrophage activation syndrome (MAS) or secondary HLH is seen when HLH presents in the setting of an autoimmune disorder such as SLE. These patients typically present with a rapid decline and development of multiorgan failure, requiring admission into the intensive care unit [9]. According to the Histocyte Society Treatment Protocol (HLH-2004), patients must meet five of the following criteria: fever, splenomegaly, cytopenias in at least two cell lines, hypertriglyceridemia and/or hypofibrinogenemia, hemophagocytosis in tissue biopsy, low or absent natural killer cell activity, elevated ferritin (>500 ng/dL), or elevated soluble CD25 (soluble interleukin-2 receptor) (>2,400 ng/dL) [10]. The typical treatment for HLH includes etoposide, dexamethasone, and cyclosporine as well as treatment of the underlying autoimmune disorder in case of MAS.

## Case presentation

A 69-year-old female with a previous admission for HLH, which was diagnosed with bone marrow biopsy, and adult-onset Still’s disease initially presented to an outside hospital with concern for sepsis in the setting of multifocal pneumonia. At the outside hospital, she was treated for septic shock with meropenem and eventually transferred to the Brooke Army Medical Center for the continuation of care.

While a bone marrow biopsy was not repeated, the clinical presentation, known previous diagnosis of HLH, and other laboratory findings suggested HLH. However, because the patient presented with an acute adult-onset Still’s disease flare, she was diagnosed with MAS. She required intubation during her admission at an outside hospital. She arrived intubated and over the first two days following the transfer was weaned and later extubated. During her hospital stay, she was continued on anakinra and decadron for treatment of her HLH/MAS. Several days after the extubation, the patient had an episode of left arm repetitive flexion and leftward deviation of eyes lasting four minutes. She was given Ativan due to continued deviation of her eyes. During this period, the patient desaturated to the 40s and her systolic blood pressure was in the 200s. Subsequently, the patient was intubated and imaging was obtained.

**Figure 1:**
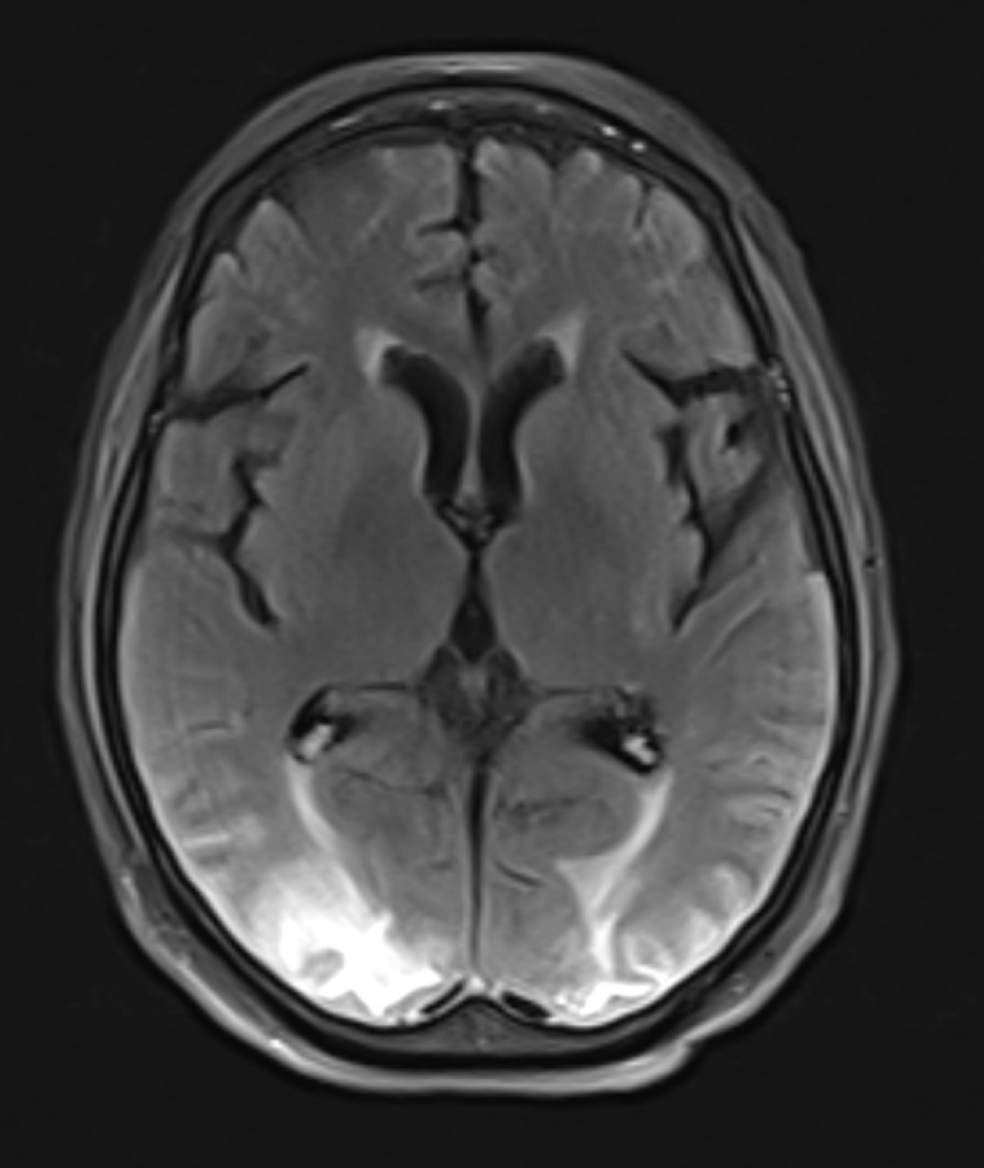
T2/FLAIR hyperintensities of bilateral occipital lobes. FLAIR: fluid-attenuated inversion recovery

**Figure 2:**
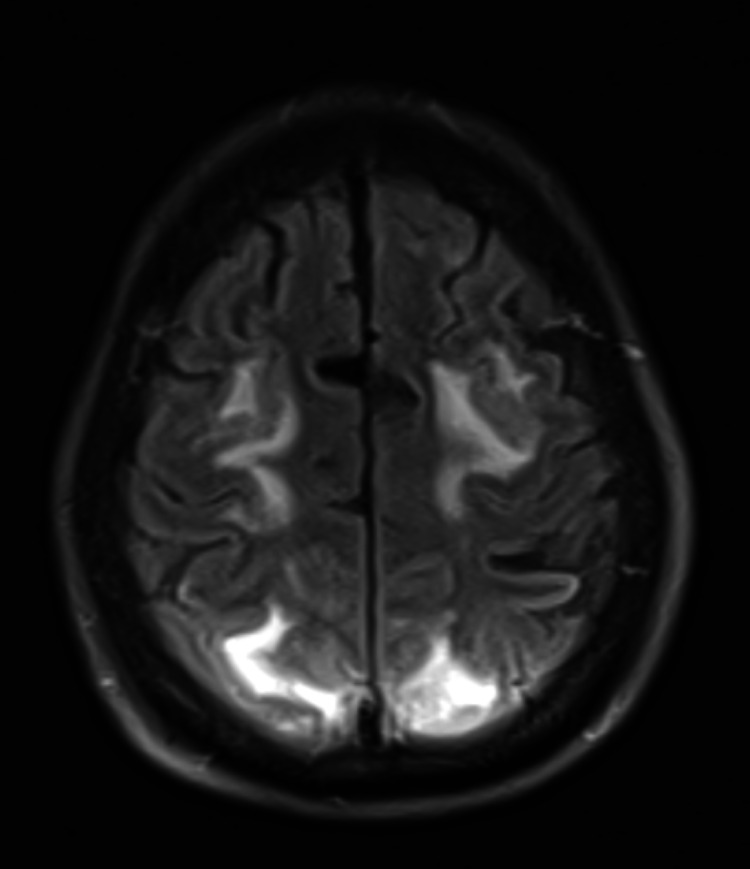
T2/FLAIR hyperintensities of bilateral ACA and PCA territories. FLAIR: fluid-attenuated inversion recovery; ACA: anterior cerebral artery; PCA: posterior cerebral artery

The initial CT head showed bilateral parietal white matter hypodensities concerning for embolic or watershed strokes or progressive multifocal leukoencephalopathy. An MRI obtained later that day demonstrated multifocal T2/fluid-attenuated inversion recovery (FLAIR) hyperintense signal abnormalities within the bilateral ACA/MCA watershed territories as well as the PCA territory with associated DWI hyperintensity, internal foci of microhemorrhages, and faint cortical enhancement suggestive of PRES, with other less likely considerations, including sequelae of subacute watershed infarct, post-inflammatory syndrome from known HLH, and less likely sequelae of hypoxic-ischemic injury. The T2/FLAIR hyperintensities can be seen in Figures 1, 2 below. The patient underwent a lumbar puncture to assess for inflammatory or infectious etiologies of the seizure. Cerebrospinal fluid testing demonstrated glucose of 68 mg/dL, protein 43 mg/L, red blood cell count of 37/µL, and polymorphonuclear cell count of 9/µL. She was also found to be herpes simplex virus 2 (HSV2) positive. She was started on Keppra twice daily for further seizure prevention which was increased following further seizure-like activity two days after the initial seizure. In addition, she was started on a 21-day course of acyclovir three times daily for the treatment of HSV encephalitis. Blood pressure was controlled using a nicardipine drip and eventual transition to oral amlodipine. Given significant medical comorbidities and worsening clinical status, the patient was eventually transitioned to comfort care and later to inpatient hospice.

## Discussion

This case represents a rare case of secondary HLH (MAS) with PRES and coinfection with HSV2. Over 95% of patients after the neonatal period are eventually diagnosed with HSV encephalitis are found to have HSV1, while HSV2 is the predominant HSV infection in the neonatal period [11]. Regardless, the pathophysiology and invasion by both viruses are very similar with a strong predilection for the medial temporal lobes. While this patient did have a positive polymerase chain reaction for HSV2, it is exceedingly rare for HSV to infect and damage the frontal, parietal, and occipital cortices while sparing the medial temporal lobe. Therefore, we believe that this may have been incidental or more likely an early reactivation without evidence of extensive infection on MRI.

As for the etiology of PRES, while immunomodulatory agents have been associated with PRES due to endothelial dysfunction, anakinra has not been implicated as a causative agent for PRES. On the other hand, there have been some small studies evaluating pediatric patients with HLH who subsequently developed significant neurologic changes and were found to have PRES. One case series involved five pediatric patients from 2004 through 2007. Four out of the five patients were found to have PRES leading the authors to suggest that pediatric patients with HLH likely have a higher risk of PRES due to the combination of the pro-inflammatory state of HLH as well as the combination of etoposide, dexamethasone, and cyclophosphamide.

Given this, there seems to be an association of HLH (and MAS) with the development of PRES, likely related to the underlying inflammatory response and autoimmunity which can be further exacerbated by medications that can induce endothelial dysfunction.

We believe that having a high level of suspicion for PRES in patients with worsening mental status associated with HLH/MAS, especially if they are being treated with immunomodulating agents, is warranted. The suspicion should be further raised if the patient develops seizures without a previous history of seizures or epilepsy. Rapid intervention with removal of the offending medication should be weighed against the treatment of the HLH/MAS as both seem to be implicated in the development of PRES.

## Conclusions

PRES is associated with many risk factors and underlying conditions that may contribute to its pathophysiology. While there is still much to learn about the complete pathophysiology of this syndrome, it appears that there is an ever-expanding list of inflammatory conditions and states and infections that may predispose a patient to PRES, including HLH/MAS and HSV2. Further, additional research is required to determine the role of immunomodulatory or immunosuppressive therapy in the precipitation and treatment of PRES.
